# Generation Pep – study protocol for an intersectoral community-wide physical activity and healthy eating habits initiative for children and young people in Sweden

**DOI:** 10.3389/fpubh.2024.1299099

**Published:** 2024-02-16

**Authors:** Matti Leijon, Albin Algotson, Susanne Bernhardsson, David Ekholm, Lydia Ersberg, Malin J-son Höök, Carolina Klüft, Ulrika Müssener, Elisabeth Skoog Garås, Per Nilsen

**Affiliations:** ^1^Generation Pep, Stockholm, Sweden; ^2^Department of Health, Medicine and Caring Sciences, Linköping University, Linköping, Sweden; ^3^Department of Learning, Informatics, Management and Ethics, Karolinska Institutet, Stockholm, Sweden; ^4^Department of Management and Engineering, Faculty of Science and Engineering, Linköping University, Linköping, Sweden; ^5^Region Västra Götaland, Research, Education, Development, and Innovation Primary Health Care, Gothenburg, Sweden; ^6^Department of Health and Rehabilitation, Unit of Physiotherapy, Sahlgrenska Academy, Institute of Neuroscience and Physiology, University of Gothenburg, Gothenburg, Sweden; ^7^Department of Culture and Society, Linköping University, Linköping, Sweden; ^8^Sahlgrenska Academy, University of Gothenburg, Gothenburg, Sweden; ^9^The Swedish Association of Local Authorities and Regions, Stockholm, Sweden; ^10^School of Health and Welfare, Halmstad University, Halmstad, Sweden

**Keywords:** physical activity, eating habits, community-wide, intersectoral, public health, implementation, children, young people

## Abstract

**Background:**

There is overwhelming evidence for the preventive effects of regular physical activity and healthy eating habits on the risk for developing a non-communicable disease (NCD). Increasing attention has been paid to community-wide approaches in the battle against NCDs. Communities can create supportive policies, modify physical environments, and foster local stakeholder engagement through intersectoral collaboration to encourage communities to support healthy lifestyles. The Pep initiative is based on intersectoral community-wide collaboration among Sweden’s municipalities. Primary targets are municipality professionals who work with children and young people as well as parents of children <18 years. The goal is to spread knowledge and create commitment to children’s and young people’s health with a special focus on physical activity and healthy eating habits to facilitate and support a healthy lifestyle. The overarching aim of the research project described in this study protocol is to investigate factors that influence the implementation of the Pep initiative in Sweden, to inform tailored implementation strategies addressing the needs and local prerequisites of the different municipalities.

**Methods:**

The project includes a qualitative and a quantitative study and is framed by a theoretical model involving four complementary forms of knowledge, explicitly recognized in the Pep initiative: knowledge about the issue; knowledge about interventions; knowledge about the context; and knowledge about implementation. Study 1 is a focus group study exploring barriers and facilitators for implementing the Pep initiative. The study will be carried out in six municipalities, selected purposively to provide wide variation in municipality characteristics, including population size and geographical location. Data will be analyzed using thematic analysis. Study 2 is a cross-sectional web-based survey investigating the implementability of the Pep initiative in Sweden’s 290 municipalities. Conditions for implementing different areas of the Pep initiative will be examined in terms of the acceptability, appropriateness, and feasibility, three predictors of implementation success. Data will be analyzed using non-parametric statistics.

**Discussion:**

The findings of the two studies will increase understanding of the prerequisites for implementing the Pep initiative in Swedish municipalities, which will provide valuable input into how implementation of the Pep initiative can best be facilitated in the different municipality settings.

## Background

### Introduction

There is an alarming worldwide growth in the prevalence of non-communicable diseases (NCDs), such as cardiovascular diseases, type II diabetes, and cancer. Noncommunicable diseases account for 74% of all deaths globally, corresponding to 41 million deaths each year, of which17 million are people under the age of 70 (1). According to the International Diabetes Federation (IDF), 537 million adults worldwide were living with diabetes in (2), a rise of 16% since the previous IDF estimates in 2019 (2). Several reports have also indicated that NCDs increasingly affect younger generations (3, 4).

Lifestyle behaviors, particularly physical inactivity and unhealthy eating habits, are strongly associated with NCDs (5–8). There is overwhelming evidence for the preventive effects of regular physical activity and healthy eating habits on the risk of developing an NCD (9). For example, a 25% reduction in physical inactivity is estimated to prevent about 1.3 million NCD-related deaths annually (10). Combining physical activity and a healthy diet can prevent a significant proportion of the 18 million deaths caused by high blood pressure, high body mass index, high fasting blood glucose, and high total cholesterol (11).

Despite the evidence base for the health benefits of lifestyle changes, research shows that it is difficult to make changes in physical activity and eating habits (12). Poor adherence to regular physical activity is a well-documented challenge among people with obesity, diabetes, heart disease, and many other health conditions. Many people also face numerous obstacles to improving their diet, e.g., lack of time to cook at home, financial issues, insufficient support from family and friends, and poor knowledge about what constitutes a healthier diet (13).

In the battle against NCDs, increasing attention has been paid to community-wide approaches to reduce the prevalence of cardiovascular diseases, diabetes, and cancer in populations. These approaches differ from traditional linear models of cause-and-effect, e.g., interventions directed at individuals and targeting a specific health problem (14). Communities can create supportive policies, modify physical environments, and foster engagement from local stakeholders to encourage whole communities to support healthy lifestyles (15, 16). Intersectoral collaboration among public and private organizations in the community is seen as a key to achieving successful community-wide initiatives (17).

### Public health in Sweden

The responsibility for public health-related issues in Sweden is shared between 290 municipalities, 21 regions (formerly known as county councils), and the state. All government levels in Sweden–national, regional, and local (municipalities) –carry out public health activities and services that affect public health. The national level issues laws, regulations, and policies, and sometimes leads specific initiatives to set the direction for activities at the regional and local levels, still local government enjoys a certain degree of autonomy in carrying out services. Several national public agencies are involved in public health activities, e.g., the Public Health Agency of Sweden, the National Board of Health and Welfare, the Swedish National Board of Housing, Building and Planning, the Swedish Food Agency, and the Swedish Medical Products Agency. Much of the national public health policy is aimed at creating societal conditions for good and equal health in the entire population and at reducing differences between different groups in structural determinants that affect public health - which also are conducive to healthy lifestyles.

The regions are mainly responsible for providing publicly funded health services, regional development, and local transport. The municipalities are responsible for the welfare of its residents, providing preschool, primary and secondary level education, care for older people and people with disabilities, social welfare, and physical planning. Both regions and municipalities make decisions about the services that are closest to their citizens, in particular concerning public health and welfare services (18). Regions and municipalities are represented at the national level by the Swedish Association of Local Authorities and Regions (SALAR), which participates on behalf of its members in discussions on policies regarding public health matters (18).

Sweden has a tradition of broad public health initiatives to support healthy lifestyles among the entire population. The overarching objective of the public health work is to achieve equitable health throughout the population, with a pronounced goal to reduce avoidable health inequalities within a generation (19). The work for the public health and welfare of the population in Sweden is often intersectoral, involving not only the regions and municipalities but also many governmental and non-governmental organizations.

### The Pep initiative

The Pep initiative was launched within Generation Pep, which is a non-governmental, non-profit organization with a vision that “All children and young people should have good opportunities to live an active and healthy life” (20). The primary targets of the Pep initiative are municipality professionals who work with children and young people, as well as parents of children under 18 years of age. The goal is to disseminate knowledge and create commitment to children’s and young people’s health, with a special focus on physical activity and healthy eating habits to facilitate a healthy lifestyle. The ambition is to challenge existing structures, cultures, and other barriers in Sweden’s 290 municipalities that might exist with regard to achieving increased physical activity and healthy eating habits among children and young people. The initiators of Generation Pep are the Crown Princess couple of Sweden and the organization is financially supported by a number of Swedish companies and foundations.

The Pep initiative is based on intersectoral collaboration in the municipalities, both informal networking and formal inter-agency collaboration among public, non-governmental, and private organizations. Therefore, the initiative involves many local actors, including municipality leadership, planning and maintenance (concerning playgrounds, green areas, forests, roads, bicycle lanes, outdoor and indoor sports and exercise facilities, etc.), preschools, schools, the region’s health services, including primary, maternity and child healthcare, non-governmental organizations, businesses, and sport and recreation associations.

The Pep initiative was inspired by a number of key themes (also called “investments”) characterizing multi-level community-wide programs that are considered most likely to influence physical activity according to the International Society for Physical Activity and Health (ISPAH) (21). ISPAH is an international organization working to advance research, policy and practice to promote physical activity and establish consensus on “what works” to achieve increased physical activity. Five ISPAH investments have inspired Pep.

#### Whole-of-school programs

It is desirable to promote physical activity to all members of the school community through supportive policies, environments, and sustainable opportunities, e.g., physical education programs that develop confidence, competence, and motivation to be active, active classrooms, and activities during recess/break times.

#### Active transport

Active transportation to and from places facilitates physical activity for many people, e.g., by improving destination accessibility, designing pedestrian-friendly and cycling-friendly infrastructure, reducing distance to public transport, and enhancing the desirability of active travel modes.

#### Active urban design

The way urban and suburban environments are built and designed matters for physical activity. It is desirable to create neighborhoods that locate shops, schools, parks, recreational facilities, jobs, and other services near homes and provide highly connected street networks that make it easy for people to walk and cycle to destinations.

#### Health care

Healthcare professionals are well positioned to promote physical activity since they meet large proportions of the population and are a trusted source of health advice. Provision of brief advice and counseling is important, particularly when linked with community opportunities and support. Brief interventions can be augmented by “physical activity on prescription,” an effective but underutilized method to increase physical activity.

#### Sports and recreation for all

Participation in sports and recreation can be encouraged through the provision of accessible and appropriate places and spaces, including both indoor and outdoor facilities as well as opportunities through formal and informal clubs and programs.

The Pep initiative has added two themes.

#### Food environment for better health

This theme recognizes that the food environment, and not just our food habits, is decisive for what we eat. The theme is inspired by the Public Health Agency of Sweden, which is developing a proposal together with the Swedish Food Agency for national goals and indicators to achieve sustainable food consumption (22).

#### Children’s perspective

This theme is inspired by the non-negotiable standards and obligations of the United Nations’ Convention on the Rights of the Child (23). The standards were adopted by the United Nations in 1989 and were ratified by Sweden in 1990 before becoming a law in 2020. The treaty sets minimum entitlements and freedoms that should be respected by governments. The four core principles of the Convention are: non-discrimination; devotion to the best interests of the child; the right to life, survival and development; and respect for the views of the child. The Convention says childhood is separate from adulthood and is a special, protected time, in which children must be allowed to grow, learn, play, develop, and flourish with dignity. The Convention is used to promote a focus on children, e.g., when municipalities plan playgrounds, schoolyards, and the physical environment of school cafeterias.

The seven themes are supplemented by national recommendations, including guidelines for physical activity and sedentary habits issued by the Public Health Agency (22) and the equivalent for food and diet by the National Food Administration (24).

Generation Pep produces a wealth of information materials of relevance for the seven themes, including various guides, checklists, and manuals, for possible use by the initiative and by the municipalities. Examples include the web-based platform Pep School, a free digital support tool for all schools in Sweden targeting employees within the educational sector. It includes an inspiration bank concerning physical activity and healthy food, focusing on six areas: school meal as an educational tool; active breaks; nutritious food in school; physical activity as a natural part of the education; pleasant school meals; physical activity in connection to the school day. A similar platform called Pep preschool is also available. Pep Park provides a framework for municipalities’ playgrounds with the aim of increasing knowledge and creating opportunities for physical activity for all children, including guidance on appropriate equipment for children with disabilities and places that invite adults to be physically active together with children. Street Pep is a week-long mobile activity festival for children and young people to try out sports and activities with healthy food. Pep Day is a cost-free activity with the goal of lowering the barriers for children to find their favorite activity and providing information about healthy eating habits and physical activity in a fun way. Another example is the book *Saga Sagor*, which has been produced for distribution in child healthcare (25). It includes inspirational tips for children and parents to be active and has been translated into Arabic, Somali and English. The book was distributed in 2019 to all five-year olds nationally, with 500,000 copies delivered thus far.

### Aim

The overarching aim of this research project is to investigate factors that influence the implementation of the Pep initiative in Sweden, with the goal to acquire knowledge to select and execute relevant strategies to support the different themes of the initiative. This is achieved through two studies which are described in this study protocol.

## Methods

### Theoretical framework

**Figure 1:**
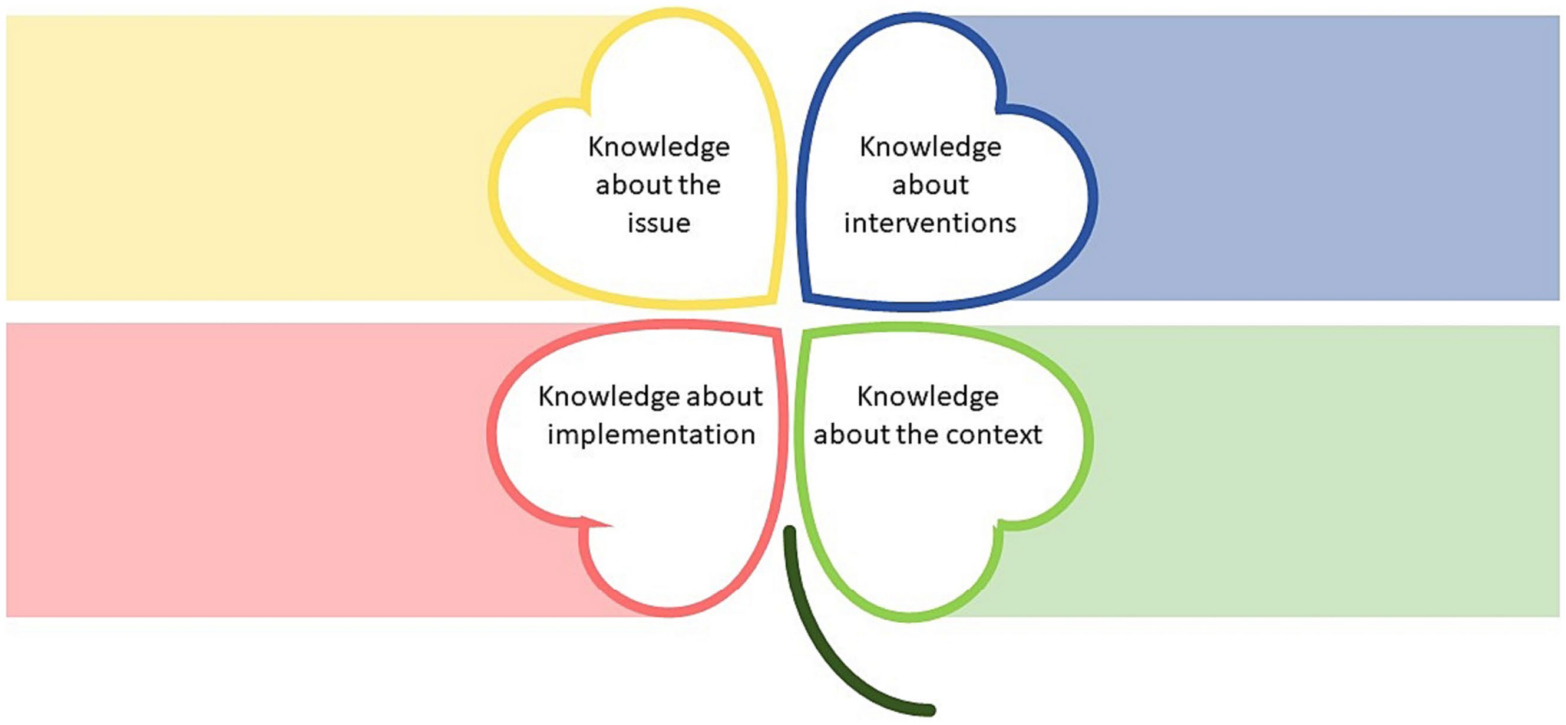
Four-leaf clover representing the four forms of knowledge informing the Pep initiative.

The research-informed Pep initiative is aimed at improving physical activity and healthy eating habits among children and young people, using an intersectoral community-wide approach. A conceptual model was developed by the authors that describes four complementary forms of knowledge, explicitly recognized in the initiative (Figure 1; Table 1). The model is intended to form a common understanding among the different stakeholders in the Pep initiative of the types of knowledge necessary to achieve the desired change, and has been used in the planning and development of the initiative as well as this research project. The four knowledge forms are visually represented by a four-leaf clover: knowledge about the issue; knowledge about interventions; knowledge about the context; and knowledge about implementation.

**Table 1:** Knowledge used in the Pep initiative.

Knowledge area	Knowledge sources used in the Pep initiative	Purpose of the knowledge
Knowledge about the issue	Guideline recommendations summarized in *FYSS* and *Nordic Nutrition Recommendations 2012: Integrating nutrition and physical activity. Part 1.* Results are also used from national and local population surveys concerning the distribution of health in the population.	To describe the distribution and determinants of physical inactivity and poor eating habits in the population and to generate knowledge concerning differences and disparities in the population, e.g., with regard to factors such as age, gender, residence and socioeconomic status.
Knowledge about interventions	ISPAH description of eight investments and guideline recommendations concerning disease prevention issued by the Public Health Agency of Sweden and the National Food Agency.	To inform the selection, planning and execution of various initiatives in the Pep initiative.
Knowledge about the context	The community readiness model and stakeholder analysis.	To assess contextual conditions for implementing the Pep initiative as input for the selection of initiatives and how they are implemented.
Knowledge about implementation	The quality implementation framework.	To provide guidance for the implementation of the Pep initiative.

ISPAH, International Society for Physical Activity and Health.

#### Knowledge about the issue

The starting point for any public health initiative is *knowledge about the issue* that will be addressed. This knowledge is often based on epidemiological and etiological research that provides input concerning the distribution and determinants of various issues which affect the health. This research can also generate knowledge concerning differences and disparities in the population, e.g., regarding factors such as age, gender, residence, and socioeconomic status.

The Pep initiative is based on current epidemiological and etiological evidence with regard to physical activity and eating habits as presented in the handbook *FYSS* (26) and *Nordic Nutrition Recommendations 2012: Integrating nutrition and physical activity. Part 1* (24). “FYSS” is an acronym that stands for “physical activity in disease prevention and treatment” in Swedish. *FYSS* is published by the Professional Associations for Physical Activity, an organization within the Swedish Society for Medicine (where also the first Swedish version of the ISPAH investments was presented). Meanwhile, *Nordic Nutrition Recommendations 2012: Integrating nutrition and physical activity. Part 1*. is published by the Nordic Council of Ministers, an official body for inter-governmental co-operation in the Nordic Region, which seeks Nordic solutions when the countries can achieve more together than by working on their own (24).

#### Knowledge about interventions

Secondly, *knowledge about interventions* is required to develop, plan and execute intersectoral community-wide initiatives. Interventions are purposive efforts to change the natural order of things or a foreseeable sequence of events (27). Intervene literally means “to come between,” from Latin *inter* (“between”) and *venire* (“to come”). Evidence of intervention effectiveness is often based on the results of stand-alone interventions that target a specific and often narrowly defined issue. This input is not always useful when addressing multiple lifestyle issues in communities using interventions that target different levels and sectors of society. Still, there is a growing evidence base pertaining to broader community-wide initiatives for physical activity and healthy eating habits (15, 21, 28).

The interventions in the Pep initiative are influenced by five ISPAH themes (21), the theme on food environment based on Nordic nutrition recommendations (24) and the joint Public Health Agency/National Food Agency proposal on sustainable food consumption (22), and the theme on the child’s perspective. An additional ISPAH theme, community-wide initiatives, is the basis for the whole Pep initiative. Research has documented positive impact of community-wide programs for increasing physical activity, particularly levels of walking and active transport (28, 29). There is also evidence showing the importance of environmental change approaches (30). Changes in built environment infrastructure, alongside media campaigns, have been shown to increase active travel physical activity (31, 32).

#### Knowledge about the context

Thirdly, *knowledge about the context* is needed due to the highly context-sensitive nature of implementing interventions and programs in communities. Accounting for the context may be particularly relevant when implementing broad, complex programs such as the Pep initiative. The term “context” is derived from the Latin *cum* (“with” or “together”) and *texere* (“to weave”). Knowledge about what happens when the Pep initiative is “woven together” with the municipalities in Sweden is important to achieve optimal results. What works well in one municipality might not work as well in others (33). The context of the Pep initiative is characterized by inter-agency and cross-sector cooperation (34), involving many different organizations. Accordingly, knowledge about how such organizations work and interact to implement interventions and programs is required for successful implementation.

The Pep initiative municipalities are encouraged to assess contextual conditions for implementation by investigating the extent to which there is community readiness for the initiative in the municipalities. The concept of community readiness refers to how prepared a community is to take action to address a particular issue, e.g., improve physical activity and eating habits. It is assumed that communities are motivated by the difference between current health situations and the desire to reach a goal. A Community Readiness model developed by Edwards et al. (35) describes community readiness in terms of nine stages: (1) No awareness (of the problem); (2) Denial; (3) Vague awareness; (4) Preplanning; (5) Preparation; (6) Initiation; (7) Stabilization; (8) Confirmation/Expansion; and (9) Professionalization. The Community Readiness model is useful for obtaining information about what strategies might be used to support or sustain a program and for identifying and engaging supportive stakeholders.

The municipalities are also advised to conduct a stakeholder analysis to explore which local actors can participate in the Pep initiative (36, 37). The municipalities are recommended to use an analytical self-assessment tool developed for public health work by SALAR (the national employers’ organization for all municipalities and regions that offers support and advice to employers). The tool recognizes that many different competencies are required in initiatives for improved public health and it is important to systematically document and identify actors with relevant knowledge and experience. The stakeholder analysis asks questions such as: Which actors are important to involve in the initiative?; What are the benefits or advantages for them?; Are there conflicts of interest and, if so, how can they be handled?; What should be the role of these actors in the initiative, e.g., decision-making, funding, participation or dissemination?

#### Knowledge about implementation

Last but not least, *knowledge about implementation* is important when putting interventions, programs and other initiatives, such as the Pep initiative, into practice and adapting them to the context in which they operate. The Latin word *implere* means to fulfil or carry into effect, which provides a basis for a broad definition of implementation research as dealing with questions concerning how to carry intentions into effect. It is widely acknowledged that implementation of interventions often produces suboptimal results, with implementation failure occurring all too frequently (38). A key lesson from this field is that evidence in and of itself is insufficient to ascertain real-world use of interventions. Thus, evidence-based and research-informed interventions, no matter how effective, do not automatically transfer from research to practice (39). Rather, strategies are needed to support implementation of interventions and broader initiatives (40).

The municipalities are also encouraged to apply the Swedish version of the Quality Implementation Framework (QIF), developed by Meyers et al. (41) and adapted by the Public Health Agency of Sweden (42). QIF is a so-called process model that details 14 types of implementation-supportive activities to be undertaken across four temporal phases to facilitate implementation. The authors behind QIF posit that “quality implementation” is “putting an innovation into practice in such a way that it meets the necessary standards to achieve the innovation’s desired outcomes” (41), p. 482. The 14 types of activities are categorized into a four-phase temporal sequence: (1) Initial considerations regarding the setting where implementation takes place (e.g., conducting a needs, resources and readiness assessments); (2) Creating a structure for implementation (e.g., developing a plan for the implementation and identifying individuals who will take responsibility for these issues); (3) Ongoing structure once implementation begins (e.g., providing assistance and feedback to the implementers); (4) Improving future applications (e.g., retrospective analysis to identify particular strengths and weaknesses that occurred during implementation) (41).

### Study 1: qualitative study of implementation barriers and facilitators in six municipalities

#### Aim, study design and setting

The aim of Study 1 is to explore barriers and facilitators for implementing the Pep initiative perceived by municipality professionals. The purpose is to understand influences on implementation of an intersectoral community-wide initiative to achieve a healthy lifestyle among children and young people through improved physical activity and healthy eating habits.

The study is designed as a focus group study and will be carried out in six municipalities which will be selected from 10 “pilot municipalities” that have already worked with various Pep activities. The 10 municipalities have expressed interest in taking part in the study. The six chosen municipalities will be purposively selected to provide wide variation in municipality characteristics, including population size and geographical location.

Data collection for the study is planned to last from autumn 2023 to spring 2024. Data analysis and manuscript writing will follow during autumn 2024.

#### Data collection and participants

Focus group discussion will be conducted with key participants in the six municipalities. These participants will have leadership responsibilities and/or personal experience of the activities involved. They will include project managers and civil servants in the six municipalities who have been involved in the local development of the Pep initiative and therefore are expected to have knowledge about what strategies are needed to overcome barriers to implementation in their municipality.

In the composition of the focus groups, consideration of both homogeneity and heterogeneity will be made: (1) participants from the same municipality will participate in a municipality-based focus group discussion, and (2) participants who represent specific Pep initiative themes in the six municipalities will be assembled in theme-based groups. This means that there will be six municipality-based focus groups, one in the project leader group, and seven theme-based group discussions.

Participants in the groups will be selected on the basis of their individual expertise and insights into what is carried out in the initiative and their holistic overview of the initiative. The participants will be municipality professionals who work actively with various aspects of the Pep initiative and have responsibility for various initiative areas in their municipalities. They have been informed about the research project and have agreed to participate in the project.

The focus group discussions will be explorative with the ambition to capture barriers and facilitators to implementing the initiative in the municipalities. They will be guided by a topic guide, allowing the participants to convey their knowledge, experience, and perceptions. The topics will be informed by the Consolidated Framework for Implementation Research (CFIR), one of the most widely used determinant frameworks in implementation science (43). Determinant frameworks describe influences, i.e., determinants, on implementation outcomes. Each type of determinant typically comprises a number of barriers (hinders, impediments) and/or facilitators (enablers). The questions will also be informed by research on municipal governance (44), cross-sector collaboration (34), political institutions (45), service design (46), and end user involvement (47).

#### Data analysis

All focus group discussions will be recorded digitally and transcribed verbatim. They will be analyzed by a research group consisting of experienced researchers with extensive knowledge in conducting qualitative research in public health and social sciences. The analytical procedure will be based on the principles for qualitative thematic analysis as described by Braun and Clarke (48). Thus, the analysis will follow a linear, yet iterative and reflective process of six phases: (1) Familiarization with the data; (2) Generating initial codes; (3) Searching for themes; (4) Reviewing themes; (5) Defining themes; and (6) Write-up. The first phase will involve reading the transcripts to ensure familiarity with the data and noting overall impressions. In the second phase, initial descriptive codes will be generated during an iterative process in which transcripts will be read and reread. The codes will then be sorted into preliminary themes in the third phase. A theme is a pattern of codes that captures something significant or interesting about the data and/or research question. In the fourth phase, the preliminary themes will be reviewed, modified, and developed. Do the themes make sense? Phase five will be the final refinement of the themes, aiming to identify the “essence” of what each theme and sub-theme is about.

### Study 2: cross-sectional survey of the implementability of the Pep initiative in Swedish municipalities

#### Aim, study design and setting

Study 2 aims to investigate the implementability of the Pep initiative. The purpose is to analyze the conditions for implementation of the seven Pep initiative themes (i.e., the five ISPAH-inspired themes and the two additional themes developed in the Pep initiative) in terms of the acceptability, appropriateness, and feasibility of each theme. The three variables are predictors of implementation success (49, 50).

The study will be based on a cross-sectional web-based survey to respondents in all 290 municipalities of Sweden. The respondents will be municipality civil servants with knowledge and experience concerning public health and health promotion issues. They will be expected to be familiar with the conditions for public health and health-promotive work in their municipality, thus being relevant participants to provide information about the implementability of the different themes of the Pep initiative. More than one participant for each municipality will be possible.

Recruitment of participants and data collection will begin in autumn 2023 and is expected to close autumn 2024. Data analysis and manuscript writing will follow during spring 2025.

#### Data collection

Data will be collected via a webbased questionnaire, based on three brief instruments repeated for each of the seven Pep initiative themes. Eligible participants will be invited via an email that will briefly explain the aim of the study and contain a link to the questionnaire. They will provide informed consent by clicking a box at the beginning of the questionnaire.

The three instruments measure acceptability, appropriateness, and feasibility of implementation (50). Acceptability is the perception among implementation stakeholders that a given intervention is agreeable, palatable, or satisfactory. Acceptability should be viewed as a dynamic construct, subject to change with experience with the intervention or the implementation process. Appropriateness is the perceived fit, relevance, or compatibility of an intervention for a given practice setting, provider, or consumer and/or the perceived fit of the intervention to address a particular issue or problem. Feasibility is the extent to which an intervention can be successfully used or carried out within a given setting (49).

The instruments Acceptability of Intervention Measure (AIM), Intervention Appropriateness Measure (IAM) and Feasibility of Intervention Measure (FIM), were developed and validated by Weiner et al. (50). They each comprise 4 items (i.e., 12 altogether) answered on 5-point Likert-type scales (response options from “completely disagree” to “completely agree”). The study will use Swedish versions of the original AIM, IAM and FIM instruments, which have been translated and cross-culturally adapted to Swedish (51). Some modifications will be needed to adapt the instruments to the Pep initiative’s seven themes. In addition to the three instruments, demographic data on respondents and key characteristics of the municipalities will be collected.

#### Data analysis

Descriptive statistics will be used to describe the characteristics of the respondents. Responses will be presented as frequencies and proportions, for each of the seven themes. Comparative analyses will be performed between municipalities, using non-parametric statistics. Municipalities will be categorized into small, medium, and large municipalities and into three geographic regions (North Sweden, Mid-Sweden, South Sweden).

### Dissemination and implementation of study findings

The study findings will be presented in open-access, peer-reviewed scientific journals, through social media channels, and at national and international conferences.

## Discussion

The Pep initiative described in this study protocol is based on an intersectoral community-wide approach and focusses on seven themes to influence physical activity and eating habits. The importance of four complementary forms of knowledge is explicitly recognized in the initiative: knowledge about physical activity and healthy eating habits; knowledge about interventions targeting physical activity and/or eating habits; knowledge about the context in which implementation of the Pep initiative will occur; and knowledge about how to implement intersectoral community-wide programs.

Intersectoral approaches are widely considered crucial to tackle difficult health-related problems and improve public health (34). These approaches are defined as collaborative initiatives which can span across many different stakeholders (37). The Pep initiative involves actions by, and interactions among, a wide range of local actors in the municipalities, which is necessary to work with the seven cross-boundary themes that form the core of the initiative.

Still, efficient intersectoral community collaboration can be difficult to achieve. A common challenge with this type of approach pertains to governance, i.e., the capacity of intersectoral collaborators to coordinate and integrate their work (52, 53). What often occurs at the higher levels of governance is the perpetuation of silo-based management of local initiatives (54). Establishing shared norms and mutually beneficial interactions is difficult to achieve as it means finding a balance between sectoral actor autonomy and interdependence (34).

The Pep initiative addresses governance issues with the recommended use of the Community Readiness model and SALAR’s stakeholder analysis to investigate the contextual conditions to establish the required governance of the initiative in a municipality. The results of these analyses provide input for decisions regarding how to create working groups with representation from different administrative municipality units and miscellaneous local actors as well as decisions concerning which themes should be prioritized.

Challenges have also been documented with the convergence capacity of intersectoral collaboration, including the capacity to define clear, joint goals in the collective interest and to co-invest in human, material and financial resources, as well as in the knowledge and skills needed to achieve these goals (54). Studies have shown that actors involved in intersectoral collaboration can have divergent interests and frames of references (45, 52). As a result, intersectoral collaboration often fails to attain common objectives (55).

The Pep initiative has taken numerous steps to overcome barriers related to convergence. The initiative has a well-defined vision and goal that are communicated to the municipalities. The focus on seven clearly described themes is also intended to provide clarity. A further strategy is the use of the QIF as a tool to support the implementation of various activities in the municipalities. QIF is a process model, which are used in implementation research and practice to provide hands-on guidance for the planning and implementation of activities (41).

Although the Pep initiative is planned as a cohesive municipality approach to improve physical activity and healthy eating habits among young people, the initiative will not be identical in all Sweden’s 290 municipalities. Due to far-reaching autonomy, municipalities in Sweden vary considerably in how they organize their operations. Implementing initiatives *in* and *with* municipalities involves challenges related to the municipalities’ different forms of organization, and intersectoral cooperation within different municipality administrations and with local actors. Such cooperation is conditioned upon numerous factors, including the local culture and tradition with regard to issues that are prioritized, resource allocation, and other issues that might be on the agenda and require attention (52).

The Pep initiative and the planned research about the initiative have many strengths. The initiative is research-informed and explicitly accounts for relevant knowledge and evidence concerning physical activity and healthy eating habits as well as which interventions are most likely to contribute to improvements in these habits, with inspiration from the research-based ISPAH themes. The initiative also recognizes the importance of the context, emphasizing the importance of assessing the community readiness and conducting stakeholder analyses. The challenges of implementation are also accounted for, with the encouragement to use QIF as a supportive tool. The use of both quantitative and qualitative research methods is a strength, providing both comprehensive data from a nationwide survey and rich data from a smaller, purposive sample in the focus group study. Well-established implementation theories are used, strengthening the validity of the findings. CFIR will inform the development of the topic guide for the focus group discussions and the analysis of the data, while AIM-IAM-FIM are validated measures of salient implementation outcomes in the survey study.

A limitation of the included studies is the lack of involvement of the intended target group, i.e., young people in the communities who are expected to be the “end users” of the Pep initiative. However, the municipalities are expected and encouraged to involve young people and/or parents in their implementation planning, to ensure that this important perspective also is considered. Recruitment of participants to the studies from a pool of municipality stakeholders involved in the Pep initiative entails a risk that mostly engaged individuals with positive attitudes will participate, which could be a threat to the credibility and trustworthiness of the study results. We will attempt to address this risk in the recruitment process, by striving for a large and varied sample in the survey, drawing from all municipalities in Sweden, and for a composition of the focus groups that encourages a variation in background among the participants. Another potential limitation is the risk for confirmation bias, i.e., a tendency in the involved researchers to process and interpret data in a way that supports their own beliefs. We will attempt to overcome this risk by involving several researchers in all analyses, both quantitative and qualitative. Credibility and trustworthiness of the qualitative findings will be enhanced by the inclusion of quotes from the participants, as well as detailed descriptions of the analysis process.

In conclusion, the Pep initiative is a research-informed endeavor using an intersectoral community-wide approach to influence physical activity and eating habits among young people in Sweden’s 290 municipalities. The initiative has the ambition to address and account for the challenges usually associated with intersectoral approaches in terms of governance and convergence issues. The findings of the two planned studies are expected to increase understanding of the prerequisites for implementing the Pep initiative in Swedish municipalities and will form the basis for continued work with the initiative, including the selection and execution of relevant strategies for the different themes. The study findings will provide valuable input into how implementation of the Pep initiative can best be facilitated in the different municipality settings. This knowledge could also be highly relevant for implementation of other public health initiatives.

## Ethics statement

The studies have received ethical approval from the Swedish Ethical Review Agency (reference number 2023-00016-01). The studies will be conducted in accordance with the local legislation and institutional requirements. All participants will receive oral and written information about the study aims and procedures and will be required to provide their written informed consent to participate prior to participation.

## Author contributions

ML: Conceptualization, Writing – original draft, Writing – review & editing. AA: Conceptualization, Methodology, Writing – original draft, Writing – review & editing. SB: Methodology, Supervision, Writing – original draft, Writing – review & editing. DE: Conceptualization, Methodology, Writing – original draft, Writing – review & editing. LE: Writing – review & editing. MH: Conceptualization, Writing – review & editing. CK: Conceptualization, Writing – review & editing. UM: Conceptualization, Methodology, Supervision, Writing – original draft, Writing – review & editing. EG: Conceptualization, Writing – review & editing. PN: Conceptualization, Investigation, Methodology, Supervision, Writing – original draft, Writing – review & editing.
